# Field evaluation of visual endoscope-assisted transcervical artificial insemination in goats: Effects on insemination time and pregnancy outcomes under tropical conditions

**DOI:** 10.14202/vetworld.2026.169-179

**Published:** 2026-01-14

**Authors:** Sarawut Sringam, Pongthorn Suwannathada, Panisara Kunkitti, Peerapat Deesuk, Awirut Wichaiwong, Patchanee Sringam

**Affiliations:** 1 Division of Theriogenology, Faculty of Veterinary Medicine, Khon Kaen University, Khon Kaen, Thailand; 2 Veterinary Teaching Hospital, Faculty of Veterinary Medicine, Khon Kaen University, Khon Kaen, Thailand; 3 Division of Physiology, Faculty of Veterinary Medicine, Khon Kaen University, Khon Kaen, Thailand

**Keywords:** artificial insemination, cervical visualization, endoscope-assisted insemination, goat reproduction, goat fertility, reproductive biotechnology, transcervical insemination, tropical goat production

## Abstract

**Background and Aim:**

Artificial insemination (AI) in goats is constrained by the complex cervical anatomy, which limits the efficiency of conventional transcervical AI (C-TCAI), particularly under field conditions. Although laparoscopic AI (LAI) achieves higher fertility rates, its invasive nature, need for anesthesia, and high operational costs limit its routine application. Visual endoscope-assisted transcervical AI (VE-TCAI) offers a minimally invasive alternative that enables real-time cervical visualization and potentially improves procedural efficiency. This study evaluated the field performance of VE-TCAI compared with C-TCAI in native–Boer crossbred goats by assessing insemination time and pregnancy outcomes.

**Materials and Methods:**

A total of 37 multiparous native–Boer crossbred does maintained on two commercial farms in northeastern Thailand were enrolled in a completely randomized field trial. Estrus was synchronized using intravaginal progesterone-releasing devices in combination with equine chorionic gonadotropin and cloprostenol sodium. Fixed-time AI was performed 48 h after device removal using frozen–thawed semen (200 million spermatozoa per doe). Does were inseminated either by C-TCAI using a vaginal speculum or by VE-TCAI using a portable visual endoscopic insemination system. Insemination time was recorded and categorized as ≤1 min or >1 min. Pregnancy was diagnosed by transabdominal ultrasonography at 45 days postinsemination. Data were analyzed using Fisher’s exact test.

**Results:**

VE-TCAI significantly improved procedural efficiency, with a greater proportion of does inseminated within 1 min compared with C-TCAI (78% vs 39%; p = 0.020). Pregnancy rates were numerically higher in the VE-TCAI group than in the C-TCAI group (45.5% vs 33.3%), although the difference was not statistically significant (p = 0.737). Overall conception rate across both methods was 37.8%, yielding an average litter size of 1.36 kids per pregnant doe. No major procedure-related complications were observed.

**Conclusion:**

Visual endoscope-assisted transcervical AI markedly reduced insemination time and facilitated easier cervical navigation under field conditions. Although pregnancy rates did not differ significantly, the consistent numerical improvement suggests potential biological relevance. VE-TCAI represents a practical, minimally invasive alternative to C-TCAI and LAI for field-based goat breeding programs, particularly in tropical production systems, warranting validation in larger multi-farm studies.

## INTRODUCTION

Artificial insemination (AI) is a widely applied reproductive technology in livestock production that supports rapid genetic improvement, enhances herd health management, and improves overall reproductive efficiency [1–3]. Goats, in particular, benefit substantially from AI because of the high economic value of genetically superior sires and the practical limitations of natural mating, including disease transmission risks and transportation constraints [2]. Despite these advantages, the application of AI in goats remains challenging due to species-specific anatomical constraints. The caprine cervix is characterized by multiple tortuous rings and folds, rendering conventional transcervical AI (TCAI) technically demanding and highly dependent on operator skill and experience. Consequently, fertility outcomes are often lower and more variable than those reported in other livestock species [4, 5].

Pregnancy rates following standard TCAI with frozen–thawed semen are generally inconsistent and typically inferior to those achieved using intrauterine semen deposition techniques [6–8]. To overcome these limitations, laparoscopic AI (LAI) has been widely adopted as the preferred method in goats, as it allows direct deposition of semen into the uterine horns and consistently yields high pregnancy rates, reaching up to 71% under optimal conditions [9]. However, LAI is inherently invasive and requires general anesthesia, surgical facilities, specialized equipment, and highly trained personnel, which collectively restrict its feasibility for routine use under field conditions [10].

As a non-surgical alternative, the Embrapa method has demonstrated that TCAI can produce favorable reproductive outcomes when successful cervical passage is achieved, with reported pregnancy rates ranging from 50% to 80% under field conditions using frozen–thawed semen [11]. Building on this concept, visual endoscope-assisted transcervical AI (VE-TCAI) integrates the practicality of conventional TCAI with real-time visual guidance analogous to that used in LAI, while avoiding surgical intervention. Endoscopic-assisted insemination techniques have been successfully applied in sheep, cats, and dogs, where direct visualization facilitates cervical navigation, minimizes tissue trauma, and improves conception rates [12–14]. In Alpine goats, VE-TCAI has achieved pregnancy rates between 57% and 83%, outperforming both natural mating and conventional TCAI [15]. A key advantage of this approach is enhanced procedural efficiency, as visual guidance reduces insemination time and minimizes operator-dependent variability.

Previous studies in small ruminants have consistently demonstrated that endoscopic or laparoscopic approaches provide superior precision of semen deposition and improved fertility outcomes compared with conventional transcervical techniques. In sheep, LAI remains the most reliable insemination method due to cervical anatomical constraints, achieving pregnancy rates exceeding 70% under optimal conditions [9, 16]. Similarly, several endoscopic-assisted techniques, including the Embrapa method and visual-guided transcervical AI, have been developed to address comparable cervical challenges in goats, resulting in improved insemination accuracy and reduced procedural difficulty [15, 17, 18]. Notably, however, no field-based study has yet compared VE-TCAI with conventional TCAI in native–Boer crossbred goats, a production-relevant genotype in tropical smallholder systems.

Despite substantial progress in AI techniques for small ruminants, important gaps remain in the practical application of these technologies under real-world field conditions, particularly in goats. LAI is widely recognized as the most reliable technique for achieving high pregnancy rates in goats; however, its invasive nature, requirement for anesthesia, surgical facilities, and specialized expertise limit its adoption in routine breeding programs, especially in smallholder and resource-limited production systems. Non-surgical alternatives, including C-TCAI and the Embrapa method, have shown variable success that is highly dependent on cervical accessibility, operator skill, and animal-related factors. Although VE-TCAI has demonstrated promising fertility outcomes in controlled or experimental settings and in specific dairy breeds, evidence supporting its effectiveness and feasibility under field conditions remains scarce.

Importantly, there is a lack of comparative field-based studies evaluating VE-TCAI against conventional TCAI in tropical environments, where heat stress, management variability, and heterogeneous genetic backgrounds may influence reproductive performance. Moreover, most previous studies have focused primarily on pregnancy rates as the sole outcome, with limited attention given to operational performance indicators, such as insemination time, procedural efficiency, and ease of cervical navigation, factors that are critical for large-scale implementation of AI services. Data are particularly lacking for native–Boer crossbred goats, which are widely used in tropical smallholder systems but differ anatomically and physiologically from intensively managed dairy breeds. Consequently, the practical value of VE-TCAI as a minimally invasive, field-ready alternative to conventional TCAI and LAI remains insufficiently validated.

In view of these knowledge gaps, the present study aimed to evaluate the practical feasibility and reproductive efficiency of visual endoscope-assisted transcervical AI in goats under field conditions. Specifically, this study sought to compare VE-TCAI with conventional TCAI in native–Boer crossbred does by assessing insemination time as an indicator of procedural efficiency and pregnancy rate as a measure of reproductive outcome. We hypothesized that real-time cervical visualization provided by VE-TCAI would facilitate faster and more accurate semen deposition, thereby reducing insemination time and improving pregnancy outcomes compared with the conventional transcervical approach. By generating field-based evidence from commercial farms in a tropical production system, this study aimed to determine whether VE-TCAI could serve as a practical, minimally invasive alternative for routine AI programs and contribute to improved reproductive efficiency in smallholder goat farming systems.

## MATERIALS AND METHODS

### Ethical approval

All experimental procedures involving animals were reviewed and approved by the Institutional Animal Care and Use Committee (IACUC) of Khon Kaen University, Thailand (Approval No. IACUC-KKU-75/66). The ethical approval was granted for the period from November 2024 to June 2025 and covered all animal handling, hormonal treatments, AI procedures, pregnancy diagnosis, and follow-up monitoring conducted during this field study.

The study was designed and implemented in strict accordance with the institutional guidelines for the care and use of animals for scientific purposes and complied with internationally accepted principles of animal welfare. All procedures adhered to the Animal Research: Reporting of In Vivo Experiments 2.0 guidelines to ensure transparent and responsible reporting of animal research. Particular attention was given to minimizing animal stress, pain, and discomfort throughout the study.

Estrus synchronization, semen collection, and AI were performed by trained veterinarians using standard veterinary practices to ensure animal safety and welfare. Restraint during insemination was limited to the minimum duration necessary, and no surgical interventions or general anesthesia were employed. Animals were closely monitored before, during, and after all procedures for any signs of distress, injury, or adverse reactions. Post insemination monitoring was conducted for at least 48 h, and no severe complications attributable to the procedures were observed.

Pregnancy diagnosis by ultrasonography was performed using non-invasive techniques by an experienced veterinarian to reduce handling time and avoid unnecessary stress. All animals were maintained under normal farm management conditions with unrestricted access to feed and water throughout the study period. Any animal showing signs of illness or compromised welfare was to be withdrawn from the study and provided with appropriate veterinary care, although no such cases occurred.

Overall, the experimental design prioritized animal welfare while ensuring scientific validity, and all procedures were conducted in full compliance with ethical standards for animal research.

### Study period and location

This field study was conducted between November 2024 and June 2025 on two commercial goat farms located in northeastern Thailand: Khon Kaen province (approximately 16.43° N, 102.83° E) and Sisaket province (approximately 15.12° N, 104.32° E). Both farms were officially certified as free from brucellosis and foot-and-mouth disease by the Department of Livestock Development, Thailand. The study areas are characterized by a tropical monsoon climate, with average ambient temperatures ranging from 26°C to 34°C and relative humidity between 60% and 75% during the study period. A short period of extreme heat was recorded in April, during which maximum temperatures reached approximately 36°C for 5–6 consecutive days.

### Experimental animals and study design

A total of 37 multiparous native–Boer crossbred does aged 2–5 years, weighing approximately 30–50 kg and having a body condition score (BCS) of 3 on a 5-point scale, were enrolled in the study. All does were 3–4 months postpartum and met the predefined inclusion criteria. Animals with a history of reproductive disorders, current illness, or poor body condition (BCS < 2.5) were excluded.

**Figure 1:**
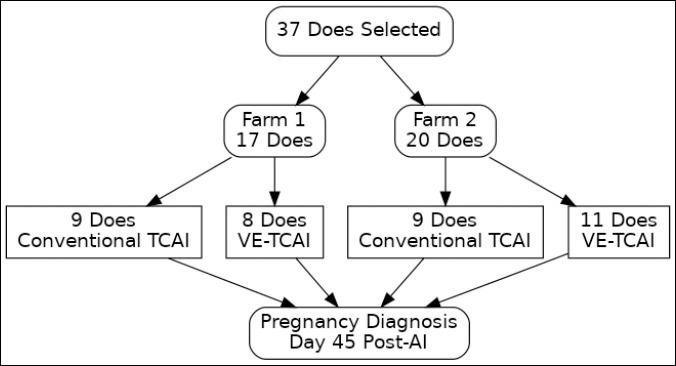
Schematic representation of animal allocation, artificial insemination (AI) methods, and pregnancy diagnosis outcomes in goats subjected to conventional transcervical artificial insemination (C-TCAI) or visual endoscope-assisted transcervical artificial insemination (VE-TCAI) under field conditions.

The experiment followed a completely randomized design. Within each farm, does were randomly allocated to one of two insemination methods, C-TCAI or VE-TCAI, using a random number generator (Figure 1). At Farm 1, nine does were assigned to the C-TCAI group and eight to the VE-TCAI group. At Farm 2, nine does underwent C-TCAI and 11 underwent VE-TCAI; unequal group sizes resulted from the limited number of eligible animals available for allocation. All animals were managed under similar conditions across farms, with free access to dry grass and water. The same veterinarian performed all inseminations and pregnancy diagnoses to minimize operator-related variation.

### Estrus synchronization protocol

Estrus synchronization was achieved using an intravaginal silicone progesterone-releasing device containing 0.3 g progesterone (CIDR®; Zoetis, USA) inserted for 13 days. On day 12, each doe received an intramuscular injection of 250 IU equine chorionic gonadotropin (eCG, Folligon®, Intervet, Germany) and 125 μg cloprostenol sodium (Estrumate®, Intervet/MSD, Germany) using 22G needles. Fixed-time AI was performed 48 h after CIDR removal. Two CIDR devices were lost during treatment, and the corresponding animals were excluded. No abnormal vaginal discharge or adverse effects were observed. Although formal estrus scoring was not applied due to the fixed-time AI protocol, behavioral signs of estrus, including vulvar swelling and clear mucus, were monitored, with approximately 20 does exhibiting visible mucus at the time of insemination.

### Semen collection and preparation

Semen was obtained from a healthy, proven fertile 4-year-old Boer buck using an artificial vagina and immediately placed in a 35°C water bath. Semen processing, evaluation, and cryopreservation followed standard protocols with minor modifications [2]. Frozen semen straws were thawed at 37°C for 30 s prior to insemination. Progressive motility was assessed at 400× magnification using a phase-contrast microscope (Olympus, Japan), and only semen batches with post-thaw progressive motility exceeding 50% were used, as this threshold is associated with acceptable fertility in field AI programs for goats [2, 9].

### AI procedures

All inseminations were performed by the same experienced operator using a single 0.25 mL semen straw containing 200 million spermatozoa to ensure procedural consistency. Prior to insemination, the vulva was cleaned and all instruments were lubricated with sterile gel. Does were restrained with their hindquarters elevated on a platform, with two assistants providing cranial and hindquarter support.

**Figure 2:**
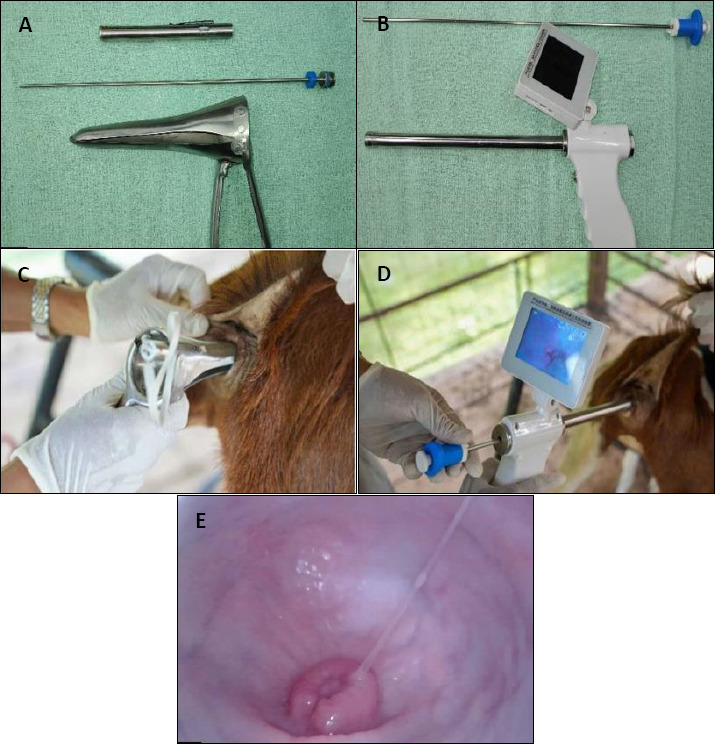
Conventional transcervical artificial insemination (C-TCAI) and visual endoscope-assisted transcervical artificial insemination (VE-TCAI) procedures in goats. (A) C-TCAI equipment comprising a stainless-steel vaginal speculum, artificial insemination gun, and light source for cervical visualization. (B) VE-TCAI system consisting of a handheld video endoscope with integrated display and insemination gun. (C) Application of the C-TCAI technique using a vaginal speculum. (D) Application of the VE-TCAI technique under real-time endoscopic guidance. (E) Endoscopic view of the goat cervix obtained using the VE-TCAI system.

For the C-TCAI method, a 17-cm vaginal speculum was used to visualize the cervix with the aid of a penlight (Figure 2a), and semen was deposited using a 25-cm goat AI gun once cervical passage was achieved. For the VE-TCAI method, a Goat Visual Insemination Gun equipped with a 22-cm probe (1-cm diameter) and a 3.5-inch LED display (resolution 640 × 480 pixels) was used (Figure 2b). A 30-cm bovine AI gun was employed for semen deposition under real-time visual guidance, and semen was released only after clear visualization of cervical canal penetration. The actual applications of the C-TCAI and VE-TCAI techniques are shown in Figures 2c and 2d, respectively, while the endoscopic view of the goat cervix obtained using the VE-TCAI system is shown in Figure 2e.

Insemination time was recorded from insertion of the instrument into the vulva to complete withdrawal and categorized as ≤1 min or >1 min. The 1-min threshold was selected as a practical benchmark based on previous evidence linking shorter insemination times with reduced cervical manipulation, lower stress, and decreased semen backflow [19, 20]. After insemination, does were maintained with elevated hindquarters for approximately 2 min to facilitate semen movement into the uterus. Instruments were cleaned, rinsed with normal saline, and disinfected in 70% isopropyl alcohol for at least 2 min between animals [21]. All does were monitored for 48 h postinsemination for adverse reactions, with none observed.

### Pregnancy diagnosis and follow-up

Pregnancy diagnosis was conducted 45 days after insemination using transrectal ultrasonography with a Honda HS-1600V scanner (Honda Electronics Co., Japan) equipped with a 7.5 MHz linear probe. All examinations were performed by a single trained veterinarian to minimize inter-observer variability. Pregnancy was confirmed by the detection of an embryonic vesicle and fetal heartbeat. Pregnant does were subsequently monitored until parturition to record kidding rate, litter size, and neonatal outcomes.

### Statistical analysis

Statistical analyses were performed using the Statistical Package for the Social Sciences version 29.0.2.0 (IBM Corp., Armonk, NY, USA) [22]. Fisher’s exact test was applied to compare insemination time categories (≤1 min vs >1 min) and pregnancy rates between insemination methods and farms. Statistical significance was set at p < 0.05.

Given the limited sample size, categorical analyses were prioritized, and effect sizes with 95% confidence intervals were calculated where applicable. The farm was treated as a random effect to account for potential environmental variation. The primary outcome measure was pregnancy status at 45 days post insemination, while secondary outcomes included insemination time, kidding rate, litter size, and adverse events. Although advanced regression modeling was not feasible, odds ratios and confidence intervals were calculated to provide quantitative estimates. Future larger-scale studies are planned to use mixed-effects logistic regression models incorporating relevant covariates, including parity, BCS, age, and post-thaw semen motility.

## RESULTS

### Insemination time and procedural efficiency

As summarized in Table 1, VE-TCAI resulted in a higher proportion of does being inseminated within 1 min compared with C-TCAI at both study sites. At Farm 1, 75.0% of does in the VE-TCAI group were inseminated within 1 min, whereas only 44.4% of does in the C-TCAI group achieved this benchmark. A similar pattern was observed at Farm 2, where 81.8% of does subjected to VE-TCAI required ≤1 min for insemination, compared with 33.3% in the C-TCAI group. When data from both farms were combined, 22 of 37 does (59.5%) were inseminated within 1 min, while the remaining 15 does (40.5%) required more than 1 min to complete the procedure.

**Table 1:** Comparison of insemination time between C-TCAI and VE-TCAI in goats under field conditions.

Farm	AI Method	No. of Does	≤1 min, n (%)	>1 min, n (%)
1	C-TCAI	9	4 (44.4%)^a^	5 (55.6%)
1	VE-TCAI	8	6 (75.0%)^b^	2 (25.0%)
2	C-TCAI	9	3 (33.3%)^a^	6 (66.7%)
2	VE-TCAI	11	9 (81.8%)^b^	2 (18.2%)
Total		37	22 (59.5%)	15 (40.5%)

AI = artificial insemination, C-TCAI = conventional transcervical artificial insemination, VE-TCAI = visual endoscope-assisted transcervical artificial insemination. Insemination time was categorized as ≤1 min or >1 min. Values are expressed as number (percentage). Different superscript letters (a, b) within the same column indicate statistically significant differences between AI methods (p < 0.05).

### Pregnancy outcomes and reproductive performance

Pregnancy outcomes following the two insemination methods are presented in Table 2. At Farm 1, pregnancy rates were comparable between the C-TCAI (33.3%) and VE-TCAI (37.5%) groups. In contrast, Farm 2 exhibited a higher pregnancy rate in the VE-TCAI group (45.5%) than in the C-TCAI group (33.3%). Overall, 14 of the 37 inseminated does (37.8%) were confirmed pregnant, resulting in the birth of 19 kids. When results from both farms were pooled, no statistically significant difference in pregnancy rate was detected between the two AI methods. The overall kidding rate was 51.4% (19 kids from 37 inseminated does), with an average litter size of 1.36 kids per pregnant doe. Twinning rates did not differ noticeably between the treatment groups. One abortion and one maternal death were recorded in the VE-TCAI group at Farm 2 during late gestation, while no stillbirths or neonatal mortality were observed in either group.

**Table 2:** Pregnancy outcomes following C-TCAI and VE-TCAI in goats.

AI Method	Farm	No. of Does	Pregnant, n (%)	Kids (n)
C-TCAI	1 2	9 9	3 (33.3%)^a^ 3 (33.3%)^a^	4 4
VE-TCAI	1 2	8 11	3 (37.5%)^a^ 5 (45.5%)^b^	4 7
Total		37	14 (37.8%)	19

AI = artificial insemination, C-TCAI = conventional transcervical artificial insemination, VE-TCAI = visual endoscope-assisted transcervical artificial insemination. Pregnancy was diagnosed by transabdominal ultrasonography at 45 days postinsemination. Values are expressed as number (percentage). Different superscript letters (a, b) within the same column indicate statistically significant differences between AI methods (p < 0.05).

### Procedure-related adverse events

Both insemination techniques were generally well tolerated under field conditions. Minor vaginal bleeding was observed in one doe from the C-TCAI group immediately after insemination; this resolved spontaneously within a few hours without intervention. No cases of cervical trauma, infection, or other procedure-related complications were detected in any of the animals across both treatment groups.

## DISCUSSION

### Comparison of insemination efficiency between AI methods

This study evaluated the comparative efficiency of C-TCAI and VE-TCAI in goats under field conditions, with a particular focus on insemination time and pregnancy outcomes. The results clearly demonstrated that VE-TCAI significantly reduced the time required for semen deposition, with 78% of does inseminated within 1 min. Statistical analysis using Fisher’s exact test confirmed the significance of this reduction (p = 0.020), supporting the hypothesis that real-time visual guidance facilitates more efficient cervical navigation. These findings are consistent with previous reports in small ruminants, as well as in cats and dogs, where endoscopic assistance improved insemination accuracy and procedural efficiency [12–15]. Shorter insemination times are advantageous under field conditions, as they reduce handling stress and may enhance fertility. In nulliparous dairy goats, the highest pregnancy rates have been observed when semen deposition occurs within 20 s [19]. From a biological perspective, reduced insemination time limits cervical manipulation and irritation, decreases semen backflow, and minimizes stress-induced uterine contractions, thereby promoting deeper and more precise semen deposition and improving the likelihood of conception [19, 20].

### Pregnancy outcomes and biological relevance

In addition to improving procedural efficiency, VE-TCAI resulted in numerically higher pregnancy rates compared with C-TCAI, with an overall conception rate of 37.8% and a notably higher rate in Farm 2 (45.5%). These outcomes align with previous studies in dairy goat breeds, where visual-guided cervical insemination achieved pregnancy rates of up to 83% when semen was deposited in the hindquarters position using a 200 μL semen volume [9], and fertility rates of 55.9% when semen was placed near the uterine body [15]. Although the overall pregnancy rate in the present field study was lower, the consistent numerical advantage observed with VE-TCAI supports the role of visual guidance in improving insemination efficiency across different goat breeds and management systems under practical farm conditions.

### VE-TCAI as a practical alternative to LAI

The present findings reinforce previous evidence that endoscopic-assisted techniques can improve AI success under variable farm conditions. VE-TCAI represents a promising non-surgical alternative to LAI, which, despite its high effectiveness, requires anesthesia, surgical facilities, and specialized expertise, thereby limiting its routine use in field operations [16, 17, 19, 23]. By shortening procedural time and achieving comparable reproductive outcomes, VE-TCAI offers a practical alternative to both LAI and conventional TCAI, which is often constrained by anatomical and technical limitations. In preliminary field trials, attempts to apply deep cervical traction similar to the Embrapa technique caused noticeable discomfort in some does, leading to the preference for VE-TCAI as a minimally invasive approach that provides direct visual guidance without cervical traction. Other reported strategies to facilitate transcervical AI include the use of smaller insemination pipettes or chemical agents to induce cervical dilation [7, 18, 24]. Additionally, the size and length of the vaginal speculum may influence the success of conventional TCAI, as inadequate dimensions can hinder cervical visualization, whereas oversized instruments may cause discomfort or trauma, ultimately affecting semen deposition accuracy [25].

### Operational feasibility and field applicability

The procedural simplicity of VE-TCAI, combined with enhanced visual guidance, enables less-experienced operators to perform AI more efficiently and accurately. From an economic perspective, VE-TCAI offers a cost-effective alternative to LAI, as it requires only a portable endoscopic system rather than surgical instruments or anesthesia. The endoscope used in this study has been in continuous operation for more than two years without technical failure, demonstrating its durability and suitability for routine field application. Although initial investment and basic operator training may represent minor barriers, VE-TCAI can be readily integrated into community-based or smallholder breeding programs as a minimally invasive, rapid, and efficient reproductive management tool. With basic restraint and limited training, AI service providers can effectively perform insemi-nations under typical field conditions. Nevertheless, strict biosecurity measures, including thorough cleaning and disinfection of endoscopic equipment between animals, are essential to prevent cross-contamination and ensure hygienic application [21].

### Factors influencing pregnancy outcomes

Despite the significant reduction in insemination time, pregnancy rates did not differ statistically between the two methods (p = 0.737), although they were numerically higher in the VE-TCAI group (45.5%) than in the C-TCAI group (33.3%). This finding suggests that factors beyond insemination duration also influence pregnancy outcomes [26]. Environmental stressors, including high ambient temperature, humidity, and nutritional fluctuations during the breeding season, may impair oocyte quality, hormonal balance, and uterine receptivity, thereby reducing conception efficiency under tropical conditions. The abortion and maternal death observed in Farm 2 may reflect the adverse effects of heat and metabolic stress on placental function and fetal survival in small ruminants [27]. Consequently, the lack of statistical significance in pregnancy rates is more likely attributable to the limited sample size rather than the absence of a biological effect, given the consistent trend toward higher fertility in the VE-TCAI group.

### Procedural efficiency as a performance indicator

Unlike earlier studies conducted in experimental stations or focused on pure breeds, this study demonstrates the feasibility of VE-TCAI under field conditions in native–Boer crossbred goats, a production-relevant genotype in tropical systems where LAI is often impractical. To the best of our knowledge, this is the first study to quantify insemination duration as an operational performance indicator in goats and to propose a ≤1-min benchmark for evaluating procedural efficiency under field conditions. The approximately 40% reduction in insemination time achieved with VE-TCAI highlights its potential as a measurable efficiency parameter in reproductive biotech-nology. Although correlation or regression analyses were not feasible due to the categorical data structure and limited sample size, the observed trend linking shorter insemination times with higher conception success suggests a mechanistic relationship between procedural efficiency and fertility outcomes. This observation is consistent with previous evidence indicating that precise intrauterine or deeper transcervical semen deposition improves fertility by enhancing sperm transport and reducing semen loss [16, 25, 28].

### Influence of estrus synchronization and timing of insemination

Estrus synchronization protocols and the timing of insemination are critical determinants of pregnancy success. In the present study, synchronization using a CIDR device combined with eCG and Prostaglandin F_2_ alpha proved effective for fixed-time AI. A 48-h interval after CIDR removal was selected based on pre-study observations and was considered to fall within the ovulatory window. Although earlier studies suggested an optimal insemination window of 56–60 h [8, 29], breed-specific physiology, environmental conditions, and hormonal regimens may have influenced ovulation timing, allowing satisfactory conception at the shorter interval. Insemination conducted 12–24 h after the onset of standing estrus is known to yield higher pregnancy rates, and multiple inseminations within the same estrus may further improve outcomes by ensuring the presence of viable spermatozoa at ovulation. Hormonal protocols incorporating eCG can enhance estrus expression and ovulation synchronization, thereby supporting AI success [30, 31].

### Limitations and future research directions

Although VE-TCAI requires a higher initial investment than conventional AI equipment, its potential to improve efficiency and reproductive outcomes may render it economically viable for medium- to large-scale goat operations [1]. Nonetheless, strict sanitation and sterilization protocols remain essential, as inadequate cleaning of endoscopic equipment increases the risk of pathogen transmission [21].

The lack of blinding during pregnancy diagnosis may have introduced observer bias, despite the use of objective ultrasonographic criteria. In addition, no adjustment for multiple comparisons was applied, which may slightly increase the risk of type I error. Future studies should incorporate larger sample sizes, blinded assessments, and multi-farm designs to validate these findings. The integration of endoscopic systems with real-time ovulation monitoring may further optimize conception outcomes in field settings.

### Broader implications for reproductive biotechnology

Beyond its immediate application in AI, VE-TCAI may serve as a foundational platform for advancing reproductive biotechnology in small ruminants. Its minimally invasive nature and real-time visual access make it suitable for future integration with non-surgical embryo transfer, genetic improvement programs, and synchronization-based breeding strategies. Furthermore, coupling VE-TCAI with real-time ovulation monitoring or AI-assisted image analysis for cervical navigation could enable precision timing and scalable reproductive management under field conditions.

## CONCLUSION

This field-based study demonstrated that VE-TCAI significantly improved procedural efficiency in goats compared with C-TCAI. Specifically, VE-TCAI reduced insemination time by approximately 40%, with 78% of does inseminated within ≤1 min, a difference that was statistically significant (p = 0.020). Although pregnancy rates did not differ significantly between methods, VE-TCAI consistently yielded higher numerical conception rates (45.5% vs 33.3%), particularly under farm conditions characterized by environmental variability. These findings indicate that real-time cervical visualization facilitates more accurate and easier semen deposition under field conditions.

From a practical perspective, VE-TCAI offers a minimally invasive, field-ready alternative to LAI that does not require anesthesia, surgical facilities, or advanced infrastructure. The marked reduction in insemination time lowers animal handling stress, improves workflow efficiency, and enhances the feasibility of large-scale or community-based AI programs. The portability and durability of the endoscopic system, combined with its ease of use, make VE-TCAI especially suitable for smallholder and tropical goat production systems where conventional LAI is impractical.

The major strengths of this study include its field-based design, evaluation under commercial farm conditions, and focus on a production-relevant genotype (native–Boer crossbred goats). This study is the first to quantify insemination duration as an operational performance indicator in goats and to propose a ≤1-min benchmark for procedural efficiency. Standardization of insemination procedures and the use of a single experienced operator minimized technical variability and strengthened the internal validity of the findings.

The study was limited by a relatively small sample size and the absence of blinding during pregnancy diagnosis, which may have reduced statistical power and introduced potential observer bias. Environmental stressors inherent to tropical field conditions, such as heat and humidity, may also have influenced reproductive outcomes and masked differences in pregnancy rates between groups.

Future studies should involve larger, multi-farm trials with diverse goat breeds and incorporate mixed-effects modeling to account for animal- and farm-level variability. Integration of VE-TCAI with real-time ovulation monitoring, repeated insemination strategies, or AI-assisted image analysis may further enhance conception outcomes. The potential application of VE-TCAI in non-surgical embryo transfer and advanced genetic improvement programs also warrants investigation.

In conclusion, VE-TCAI represents a practical, efficient, and minimally invasive alternative to C-TCAI and LAI methods. By significantly reducing insemination time and demonstrating favorable reproductive trends under field conditions, VE-TCAI provides a scalable pathway to improve reproductive efficiency and genetic progress in goats, particularly in resource-limited and smallholder farming systems.

## DATA AVAILABILITY

The datasets generated during the current study are available upon reasonable request from the corresponding author. There were no deviations from the approved experimental protocol during the study.

## AUTHORS’ CONTRIBUTIONS

SS: Conceptualized the study and revised the manuscript. SS, PD, and AW: Investigation and data collection. SS and PSu: Data analysis and interpretation were conducted. PK and PSr: Provided technical guidance and reviewed the manuscript. All authors have read and approved the final version of the manuscript.
